# Electroencephalography Correlates of Well-Being Using a Low-Cost Wearable System

**DOI:** 10.3389/fnhum.2021.745135

**Published:** 2021-12-24

**Authors:** Cédric Cannard, Helané Wahbeh, Arnaud Delorme

**Affiliations:** ^1^Centre de Recherche Cerveau et Cognition (CerCo), Centre National de la Recherche Scientifique (CNRS), Paul Sabatier University, Toulouse, France; ^2^Institute of Noetic Sciences (IONS), Petaluma, CA, United States; ^3^Swartz Center for Computational Neuroscience (SCCN), Institute of Neural Computation (INC), University of California, San Diego, San Diego, CA, United States

**Keywords:** wearable EEG, alpha asymmetry, frontal, temporoparietal, executive control, well-being, large sample analysis

## Abstract

Electroencephalography (EEG) alpha asymmetry is thought to reflect crucial brain processes underlying executive control, motivation, and affect. It has been widely used in psychopathology and, more recently, in novel neuromodulation studies. However, inconsistencies remain in the field due to the lack of consensus in methodological approaches employed and the recurrent use of small samples. Wearable technologies ease the collection of large and diversified EEG datasets that better reflect the general population, allow longitudinal monitoring of individuals, and facilitate real-world experience sampling. We tested the feasibility of using a low-cost wearable headset to collect a relatively large EEG database (*N* = 230, 22–80 years old, 64.3% female), and an open-source automatic method to preprocess it. We then examined associations between well-being levels and the alpha center of gravity (CoG) as well as trait EEG asymmetries, in the frontal and temporoparietal (TP) areas. Robust linear regression models did not reveal an association between well-being and alpha (8–13 Hz) asymmetry in the frontal regions, nor with the CoG. However, well-being was associated with alpha asymmetry in the TP areas (i.e., corresponding to relatively less left than right TP cortical activity as well-being levels increased). This effect was driven by oscillatory activity in lower alpha frequencies (8–10.5 Hz), reinforcing the importance of dissociating sub-components of the alpha band when investigating alpha asymmetries. Age was correlated with both well-being and alpha asymmetry scores, but gender was not. Finally, EEG asymmetries in the other frequency bands were not associated with well-being, supporting the specific role of alpha asymmetries with the brain mechanisms underlying well-being levels. Interpretations, limitations, and recommendations for future studies are discussed. This paper presents novel methodological, experimental, and theoretical findings that help advance human neurophysiological monitoring techniques using wearable neurotechnologies and increase the feasibility of their implementation into real-world applications.

## Introduction

### Well-Being

The question of what determines well-being has intrigued humans throughout recorded history and to this day remains a topic of significant interest and debate (Dodge et al., 2012; Alexandrova, 2015). The hedonic view of well-being focuses on the emotional dimension (i.e., positive and negative affect) to address this question. The eudaimonic perspective focuses on the sense of striving toward realizing one’s potential or goals, a life purpose, and seeking personal growth (Ryan et al., 2008). Well-being is now understood as a multidimensional and dynamic construct encompassing both views and other new essential components such as mental and physical health, autonomy, social relationships, spirituality, self-acceptance (Keyes and Waterman, 2003; Ryff and Singer, 2013). Well-being can be mediated by numerous factors such as demographics (Keyes and Waterman, 2003; Carstensen et al., 2011), genetic predisposition (Keyes et al., 2010), personality traits (Lucas and Diener, 2008), income (Luhmann et al., 2011), exercise (Hassmén et al., 2000; Svensson et al., 2021), mindfulness meditation practice (Carmody and Baer, 2008), or connectedness with nature (Howell et al., 2011; Russell et al., 2013). These factors also shape the structure and function of our brains throughout the lifespan, with important implications for well-being levels. While progress has been made recently regarding our understanding of the relationships between well-being and the brain, much is still unknown (Dolcos et al., 2018). By identifying the neural correlates of well-being, we may better understand the mechanisms that underly higher levels of well-being, and in turn, develop promising interventions aiming at helping people live happier and more successful lives (Dolcos et al., 2018).

### Frontal Electroencephalographic Asymmetry

#### Definition and Calculation

For decades, frontal electroencephalographic (EEG) asymmetry has been a useful tool to study emotion-related states and traits, motivation, temperament, cognitive control, and psychopathologies (Coan and Allen, 2003, 2004; Allen et al., 2004; Harmon-Jones et al., 2010; Scherer and Ekman, 2014; Allen and Reznik, 2015; Smith et al., 2017). Frontal EEG asymmetry refers to a relative difference in alpha power spectral activity (8–13 Hz) between the left and right frontal regions of the brain. Because alpha oscillations are known to functionally inhibit regional cortical activity (Laufs et al., 2003, 2006; Oakes, 2004; Mathewson et al., 2011; Scheeringa et al., 2012; Grimshaw and Carmel, 2014), authors have associated an increased alpha activity with a decrease in brain activity or a decrease in allocation of cortical resources in the same region (Davidson, 1988; Davidson et al., 1990; Gevins et al., 1997; Cook et al., 1998; Allen et al., 2004). Thus, positive asymmetry scores (i.e., greater alpha power in the right frontal area relative to the left) are thought to reflect relatively lower right than left frontal cortical activity, and vice versa for a negative asymmetry score.

#### The Main Models

Decades of work using the alpha asymmetry metric have led to emotional valence and motivation models (Allen et al., 2004; Harmon-Jones et al., 2010). These models highlight that approach motivation emotional processes are associated with relatively greater cortical activity in the left frontal area compared to the right, which in turn, is inversely correlated with alpha power (i.e., greater right than left alpha power in these areas). Inversely, emotional processes related to avoidance motivation and a negative valence are associated with relatively greater right than left frontal cortical activity (corresponding to greater left than right frontal alpha power). Extreme approach-oriented traits and behaviors include for example positive urgency (i.e., the tendency toward rash action in response to extreme positive emotional states (Tomarken and Davidson, 1994), sensation-seeking (Santesso et al., 2008), and high reward sensitivity (Pizzagalli et al., 2005), whereas avoidance-related traits and behaviors include depression and anxiety (Thibodeau et al., 2006), shy temperament (Fox et al., 1995), negative dispositional affect (Tomarken and Davidson, 1994), and poor regulation of negative emotions (Jackson et al., 2003). These models align with the clinical literature showing that lesions in the left frontal area are associated with depression symptoms (Robinson and Price, 1982; Harmon-Jones et al., 2010).

#### The Underlying Brain Networks and Systems

Going one step further beyond these descriptive models, investigators using EEG source-localization techniques (Laufs et al., 2003; Pizzagalli et al., 2005; Mantini et al., 2007; Koslov et al., 2011; Gable et al., 2015; Smith et al., 2018) found that frontal asymmetries originate from the dorsal frontoparietal network (dFPN), the inferior frontal gyrus, and the right dorsolateral prefrontal cortex (dlPFC; which is part of the dFPN). These results led them to suspect that frontal asymmetries reflect the integrity of the supervisory system, which is theorized to generate effortful constraint and self-control (Sutton and Davidson, 1997; Cacioppo et al., 2007; Gable et al., 2015). Gable et al. (2015) suspected that the alpha asymmetry is driven by the activity of this supervisory control system, supposedly located in the right frontal area (Gable et al., 2015). Frontal asymmetries may also reflect other associated executive control mechanisms, which play an essential role in allocating attention toward a goal and inhibiting interference from distractors (Corbetta et al., 2008; Vossel et al., 2013; Grimshaw and Carmel, 2014; Gable et al., 2015). In this view, termed the asymmetric inhibition model, mechanisms in the left frontal cortex would inhibit negative distractors, whereas mechanisms in the right frontal cortex would inhibit positive distractors. Consequently, asymmetric aberrations in these systems result in bottom-up and top-down dysfunction, such as the difficulty in disengaging attention from negative/avoidance-motivation information AS in depression and anxiety (Eysenck et al., 2007; Shackman et al., 2009; Cisler and Koster, 2010; De Raedt and Koster, 2010; Engels et al., 2010; Gotlib and Joormann, 2010; Kim et al., 2012; Gable et al., 2015), whereas difficulty in inhibiting positive/approach-motivation distractors results in addiction and positive urgency behaviors (Bechara, 2005; Garavan and Hester, 2007; Goldstein and Volkow, 2011). Thus, multiple lines of research demonstrate that the mechanisms underlying alpha asymmetry measurements are highly implicated in processes that contribute to well-being (positive/negative affect, capacity to fulfill one’s potential and life goals, etc.).

### Limitations in Electroencephalographic Asymmetry Research

While the literature on EEG asymmetry is sizable and robust, it is important to note that there have also been failed replications and contradictory results (Gotlib, 1998; Reid et al., 1998; Hagemann et al., 1999; Müller et al., 1999; Coan et al., 2001; Gale et al., 2001; Papousek and Schulter, 2002; Dennis and Solomon, 2010; Stewart et al., 2010; Kop et al., 2011; Koslov et al., 2011; Quinn et al., 2014; Meyer et al., 2015; Arns et al., 2016; Palmiero and Piccardi, 2017; van der Vinne et al., 2017). These inconsistencies can be explained by the heterogeneity in the experimental designs, EEG preprocessing techniques, and statistical approaches employed across investigators over the years (Allen et al., 2004; Smith et al., 2017). A summary of the main limitations of EEG asymmetry research and proposed solutions that were implemented in this study are now described.

#### Trait Versus State

One limitation is that EEG asymmetry can reflect trait or state aspects and thus, designing experiments to highlight one over the other depending on the research question is essential. When measured during rest, EEG asymmetry is considered a trait variable related to various psychological constructs and predictive of future emotional behavior or psychopathology (Wheeler et al., 1993; Davidson, 1994; Sutton and Davidson, 1997; Stewart et al., 2010; Nusslock et al., 2011; Papousek et al., 2012). When measured as an event-related response, it is considered a state variable reflecting the person’s current emotional state (Coan et al., 2001; Harmon-Jones and Sigelman, 2001; Harmon-Jones, 2004). Some authors estimate that 60% of the variance in asymmetry measure within a resting session is due to trait influence, and the 40% to state influences (Hagemann et al., 2002). Hence, the first approach aims to reduce the state influence during rest, whereas the second one aims to increase it using emotion-elicitation perturbations (Coan et al., 2006). In this study, we focus on the trait variable and hypothesize that trait frontal alpha asymmetry will be associated with multidimensional well-being (since well-being is driven by both emotional valence and motivational components).

#### Sample Characteristics

The second limitation to EEG asymmetry research is that sample-specific characteristics (e.g., age, gender) have been shown to significantly influence EEG findings because of functional and anatomical differences (Klimesch, 1999; Sowell et al., 2007; Hagemann et al., 2008; Finley et al., 2020). Many EEG asymmetry studies include participants of one gender to reduce this bias (Tomarken et al., 1990; Wheeler et al., 1993; Jacobs and Snyder, 1996; Reid et al., 1998; Gale et al., 2001; Dennis and Solomon, 2010; Mikolajczak et al., 2010; Koslov et al., 2011). However, this prevents investigators from examining gender as a potential mediator or moderator of asymmetry findings (MacKinnon et al., 2013). There is a lack of consensus regarding the role gender plays in EEG asymmetry in the limited studies that have addressed this question (Veldhuizen et al., 1993; Carrier et al., 2001; Miller et al., 2002; Otero et al., 2003; Morgan et al., 2005; Gasbarri et al., 2006, 2007; Stewart et al., 2010; Kovacevic et al., 2015; Müller et al., 2015; Hashemi et al., 2016). Similarly, the role age plays in EEG asymmetry is also not very well known. One solution to the lack of understanding of if and how demographic variables influence EEG asymmetry and well-being is to collect large and diversified datasets that better reflect the general population. A few studies with large samples found that age and gender mediate frontal asymmetry but that ethnicity or socioeconomic status did not (Stewart et al., 2010; Gable et al., 2015; Arns et al., 2016). However, these studies are rare and hard to replicate because of the time and cost involved in recording EEG data on a large number of subjects with conventional systems (equipment cost, EEG preparations time, participants compensation for their time, equipment cleaning, etc.).

Wearable EEG technologies make the collection of large datasets of diversified and under-represented populations more feasible and offer promising new applications for both clinicians and researchers in the long term (Cannard et al., 2020). These applications include brain monitoring in naturalistic settings and in real-time (Hu et al., 2015; Jebelli et al., 2017), brain-computer interfaces (BCI; Park et al., 2020), neurofeedback interventions (Angelakis et al., 2007; Quaedflieg et al., 2016; Brandmeyer and Delorme, 2020a), neuromarketing (Cartocci et al., 2018; Ramsøy et al., 2018), or neuroaesthetics research (i.e., the science studying the biological underpinnings of aesthetic experience; Cheung et al., 2019; Cartocci et al., 2021). While these EEG systems can have inferior hardware capacities than conventional ones, recent technological and algorithmic advancements make the detection and measurement of mental states increasingly reliable (Wu et al., 2017), with as few as a single EEG channel (Umar Saeed et al., 2018; Arpaia et al., 2020; Mahmoodi et al., 2021). Additionally, these systems can easily combine other physiological measures such as electrocardiography (ECG) or galvanic skin response (GSR) to improve the efficacy of mental states detection (e.g., Ahn et al., 2019). Wearable EEG systems have been used extensively over the past few years to measure frontal asymmetry (Peng et al., 2011; Hu et al., 2015; Hashemi et al., 2016; Jebelli et al., 2017, 2018; Wu et al., 2017; Zhao et al., 2017; Hwang et al., 2018; Umar Saeed et al., 2018; Cao et al., 2019; Arpaia et al., 2020; Park et al., 2020; Saeed et al., 2020) and were used in this present study to enable the collection of a large dataset. Hence, in this study, we aim to evaluate the potential relationship between well-being, alpha asymmetry, and individual characteristics (namely age and gender) in a large sample, collected using a low-cost wearable EEG headset.

#### Alpha Frequencies and Bounds

The third main limitation in EEG asymmetry research is the handling of alpha-band frequencies and bounds. The alpha band is dominantly considered as a single phenomenon in EEG asymmetry studies. However, previous evidence suggested that it should not. For instance, measuring alpha power spectral density (PSD) on the traditionally *a priori*-defined bandwidth 8-13 Hz does not account well for interindividual differences because parts of the alpha power distribution fall outside this range for some individuals (Klimesch et al., 1990; Klimesch, 1997). Furthermore, differential changes in opposing directions within the same dataset have been observed between lower (8–10.5 Hz) and upper (11–13 Hz) alpha oscillations, as well as between local and global properties (Klimesch, 1999; Nunez et al., 2001; Nunez and Srinivasan, 2006).

The individual alpha frequency (IAF) refers to the dominant frequency within the alpha power distribution and is thought to reflect the dominant neural circuits that generate alpha oscillations. Because it varies within and across individuals, measuring alpha power on each individual’s IAF better accounts for inter-individual variability (Klimesch, 1999; Haegens et al., 2014; Mierau et al., 2017). Individual alpha frequency estimates are considered a trait-like characteristic of the human EEG (Grandy et al., 2013), have high heritability (Smit et al., 2006), decrease with age (Klimesch, 1997; Corcoran et al., 2017; Finley et al., 2020), and have good test-retest reliability (Näpflin et al., 2007). Few studies have investigated EEG asymmetry using IAF estimates to our knowledge (Klimesch et al., 1998; Angelakis et al., 2004a; Vecchiato et al., 2012; Quaedflieg et al., 2015, 2016; Di Flumeri et al., 2016).

The first approach to estimate IAF is to use the peak alpha frequency (PAF; frequency within the alpha band with the highest power). While this technique has been extensively used for the study of cognition (Klimesch, 1999; Angelakis et al., 2004b; Rathee et al., 2020), it does not perform well with a portion of the population that have ambiguous alpha peaks, “split peaks” (i.e., several peaks within the alpha band), or no peak at all (Anokhin and Vogel, 1996; Chiang et al., 2008, 2011). A second approach called the alpha center of gravity (CoG) considers the shape of the alpha PSD distribution and is thought to provide a more accurate summary of the underlying alpha activity. Initial techniques to estimate IAFs relied on visual and manual inspection (Klimesch et al., 1990) or cross-frequency assumptions (Doppelmayr et al., 1998; Klimesch, 1999; Posthuma et al., 2001; Goljahani et al., 2012). These methods were very time-consuming and prone to subjective judgment error. Novel automated methods have now been developed to avoid these limitations. While the channel-based method (CRB; Goljahani et al., 2012, 2014) is better suited for event-related EEG asymmetry, other statistical curve-fitting and clustering techniques are particularly promising for IAF-estimation of resting EEG data (Chiang et al., 2008, 2011; Lodder and van Putten, 2011, 2013; Van Albada and Robinson, 2013; Corcoran et al., 2017). Corcoran et al. (2017) have implemented these algorithms into a fast, reliable, open-source toolbox operating in MATLAB and Python (Corcoran et al., 2017). This method seems suitable for large datasets with a relatively low signal-to-noise ratio (SNR) acquired with a wearable dry EEG system.

Hence, calculating alpha asymmetry scores on PSD estimated on the predefined alpha band (8–13 Hz), the lower (8–10.5 Hz) and upper (11–13 Hz) alpha sub-bands, and the CoG may help us understand more about the underlying mechanisms of alpha asymmetry. The present study incorporates these metrics to evaluate differences in these measures and their relationship to well-being. We expect well-being to be positively correlated with CoG values, differently correlated with lower and upper alpha (no specific direction is hypothesized), and positively correlated with CoG-asymmetry (and we expect this association to be stronger than that with the traditional whole alpha band asymmetry, by better accounting for interindividual differences).

#### Limiting Electroencephalographic Asymmetry to the Frontal Areas

The fourth limitation is the reduction of the study of EEG asymmetry phenomenon to only the frontal areas. It has been expressed for a long time that both anterior and posterior cortical regions show asymmetric activity patterns (Davidson, 1988, 1992). This is also reflected by studies showing that FAA obtained on data referenced with the current-source density (CSD) transformation (i.e., reflective of alpha power from local frontal sources only) correspond to a marker for depression risk, whereas FAA obtained on data referenced to mastoids or average (i.e., containing alpha power from distal, posterior cortical regions) correspond to a better marker of current depression severity (Stewart et al., 2010). Furthermore, expanding the analysis of alpha asymmetry to the temporoparietal (TP) regions seems particularly relevant since alpha asymmetries were source-localized to the frontoparietal network (FPN), which includes brain structures in both the frontal and the TP regions (see above; Vossel et al., 2013). Furthermore, different subtypes of anxiety disorders are differently associated with asymmetric activity in frontal and TP regions (Heller et al., 1997; Engels et al., 2007; Mathersul et al., 2008; Müller et al., 2015). Together, these findings suggest that anxious arousal (physiological arousal and hyper-reactivity under conditions of panic) is associated with relatively greater right than left frontal activation, whereas anxious apprehension (involving worry and verbal ruminations; i.e., trait anxiety and generalized anxiety disorder) is linked to the opposite asymmetry in frontal area and asymmetry in the same direction in the TP area. However, other findings suggested that TP asymmetry was less stable over time compared to frontal asymmetry (Müller et al., 2015) and sometimes not associated with self-reported measures of affect and motivation (Davidson et al., 1990). In this study, we examine the relationship between well-being and asymmetry in both frontal and TP regions and hypothesize that alpha asymmetry in both regions will be associated with well-being (with potentially a different direction).

#### Limiting Electroencephalographic Asymmetry to the Alpha Oscillations

The Fifth and last main limitation in EEG asymmetry research is the need to expand analyses to other frequency bands. Coherence in both alpha and theta oscillations has been highlighted during both relaxation and mental calculation (Nunez and Srinivasan, 2006). This widespread (global) phase coherence phenomenon increases in the upper frequencies of both alpha and theta bands while it simultaneously decreases in the lower frequencies (Wingeier, 2000; Nunez and Srinivasan, 2006). These findings go along with other findings indicating that global alpha and theta rhythms functionally interact during both relaxation and attentional tasks (Klimesch, 1999; Buzsáki, 2006; Laufs et al., 2006). Furthermore, theta power has been used to predict response to depression treatment in several studies (Knott et al., 1996, 2000; Cook and Leuchter, 2001; Cook et al., 2002; Bares et al., 2008; Iosifescu et al., 2009; Spronk et al., 2011; Baskaran et al., 2012; Olbrich and Arns, 2013). Furthermore, theta power decreases while upper alpha power increases in several conditions (i.e., the early part of life until adulthood, in neurological disorders, and the transition phase from awake to sleeping), whereas the direction of their relationship is opposite for the late part of the lifespan (Klimesch, 1999).

Similarly, alpha and beta spectral power have been found to interact (Laufs et al., 2006; Hamid et al., 2010), and both are associated with high levels of mental stress and depression (Hayashi et al., 2009; Alonso et al., 2015; Jena, 2015; Al-shargie et al., 2016; Jun and Smitha, 2016; Díaz et al., 2019; Al-Dabass, 2020; de Hemptinne et al., 2021). More specifically, prefrontal beta power in lateral areas was found to be positively associated with depression and anxiety, whereas lateral beta power was negatively associated with mood (de Hemptinne et al., 2021). The authors interpreted these results to be in line with the organization of the reward networks in the prefrontal cortex (PFC).

However, no robust literature is available to make specific interpretations about how alpha asymmetry interacts with other frequency bands, and whether asymmetries in other frequency bands could be associated with psychological constructs such as well-being. Thus, we aim to bring light to this matter in this study and hypothesize that well-being will be associated with asymmetries in other frequency bands. This study includes asymmetry scores estimated on the delta (1–3 Hz), theta (4–7 Hz), and beta (14–30 Hz) frequency bands, for both frontal and TP sites. Since no previous research exists on this matter, we have no specific hypothesis concerning the direction of these potential associations.

#### Summary of the Study Goals and Hypotheses

Considering the potential importance of alpha asymmetry as a physiological correlate in general, and for well-being specifically, the overall objective of this study was to determine whether a low-cost wearable EEG headset (the Muse by Interaxon) could be used to measure EEG correlates (CoG, EEG asymmetry) of well-being on a relatively large sample (*N* = 353). The analyses were designed to address the main limitations of EEG asymmetry research addressed above. The hypotheses for the study were as follows:

1. Well-being will be positively associated with approach-motivation processes and positive valence of emotion, as reflected by relatively greater right than left alpha power. We hypothesize that this will be the case for both frontal and temporoparietal (TP) areas (although the direction might be different, based on the literature discussed).
2. Age and gender will be associated with both well-being and mean alpha asymmetry (predefined 8–13 Hz band).
3. The CoG will be positively correlated with well-being levels.
4. Asymmetry scores estimated on sub-components of alpha oscillations (namely lower/upper alpha and CoG) will provide stronger correlations regarding the relationship between well-being and alpha asymmetry than those estimated on the predefined alpha aband (8–13 Hz), by better accounting for alpha source differences (lower/upper alpha) and interindividual differences (CoG).
5. Well-being levels will be associated with asymmetries in other frequency bands (namely delta, theta, and beta), although we do not have specific hypotheses regarding which bands and their directions.

## Materials and Methods

### Participants

353 participants were recruited from groups attending workshops focusing on well-being and personal development at the Earthrise Campus. Exclusion criteria: people younger than 18 years of age, inability to read or understand the consent form, acute or chronic illness precluding completion of measurements. Upon arrival at the research laboratory, participants were briefly interviewed by the research assistants to ensure they met the inclusion/exclusion criteria and were then allocated to a carrel where the following equipment was available for their participation: a wearable EEG headset, a Chromebook, and a pair of headphones. The settings allowed the recording of up to 9 participants simultaneously. Participants volunteered and were not compensated for participation. The study and the consent form were approved by the Institute of Noetic Sciences’ institutional review board (IRB). All questionnaires were optional and anonymous.

### Multidimensional Well-Being

Participants’ multi-dimensional well-being was assessed on-site using the Arizona Integrative Outcomes Scale (AIOS; Bell et al., 2004) in SurveyMonkey^1^. The AIOS is a horizontally displayed scale that provides a quick and accurate assessment of the participants’ self-rated global sense of physical, social, psychological, affective, and spiritual well-being over the past 24 h (Bell et al., 2004). The low anchor is “Worst you have ever been” (AIOS score = 0) and the high anchor is “Best you have ever been.” (AIOS score = 100). The 24-h AIOS score was found to significantly reflect psychological well-being, global health, psychological distress, the positive and negative affect, and the positive states of mind, and was significantly correlated with the 1-month AIOS scores (Bell et al., 2004; Otto et al., 2010; Tuason et al., 2021). Furthermore, AIOS-24 h was found to be associated with personality traits (Wahbeh et al., 2021). While these findings suggest the AIOS-24 h reflects trait components of well-being, validation of this hypothesis requires further testing. The online survey included additional questionnaires that are not included in this study and are reported elsewhere (Wahbeh et al., 2021).

### EEG

#### Data Collection

Once participants completed the survey, continuous EEG was recorded using InteraXon’s Muse wearable EEG headband (version 2016). Electroencephalography data were recorded while participants were instructed to focus their attention on their breath and count inhalation/exhalation cycles. They were instructed to bring their attention back to their breath and start counting again if they lost track of their count or noticed their minds wandered. This task reduces EEG artifacts occurring naturally with eye movements. Most importantly, this task can later be implemented into practical translational and therapeutical applications aimed at increasing well-being levels through the modulation of alpha asymmetry and the underlying brain processes (Angelakis et al., 2007; Sessa, 2007; Moynihan et al., 2013; Doll et al., 2016; Schmalzl et al., 2018; Prpa et al., 2020). Electroencephalography data were with a sampling rate of 256 Hz and 12-bits of data resolution. This system has five active dry electrodes: two frontal silver (AF7 and AF8), two temporoparietal (TP) silicone electrodes (TP9 and TP10), and a reference electrode (FPz). Before positioning the headband on the subjects’ heads, their skin was cleaned with alcohol swipes at electrode sites, and a thin layer of water was applied with a sponge to the electrodes to improve signal quality. EEG data were acquired on Chromebooks using the Muse Monitor App and were uploaded onto Dropbox at the end of the recording. Random unique identifiers were used to link survey and EEG data. Impedance check was provided by the App (horseshoe symbol) and visually confirmed by the raw signal displayed on the screen in real-time.

As shown in previous publications, good internal consistency reliability of frontal EEG asymmetry can be obtained with as few as 100 epochs, corresponding to one to 3 min of artifact-free recorded data [depending on window size; (Allen et al., 2004; Towers and Allen, 2009; Smith et al., 2017)]. Allen et al. (2004) found that the number of epochs used to estimate the asymmetry scores matters more than the number of minutes of data (Allen et al., 2004), with asymmetry scores estimated on 2 min of data showing similar consistency reliability than those obtained on 8 min of data. Furthermore, a recent publication showed that individuals can robustly be differentiated using spectral EEG data obtained on segments as short as 30 s (and this was stable weeks later; da Silva Castanheira et al., 2021). Thus, 2 min of EEG data were recorded for each participant. When less than 8 min of data is available, Allen et al. (2004) recommend reporting the internal consistency reliability and how many blocks were treated through the calculation of Cronbach’s alpha (see below).

#### Data Preprocessing

Data preprocessing was done in *EEGLAB* version 2020.0 (Delorme and Makeig, 2004) in *MATLAB* v2020a. EEG data were imported with the *muse_monitor* plugin v3.2, low-pass filtered at 30 Hz (transition bandwidth 12.5 Hz; passband edge 50 Hz; cutoff frequency -6 dB 56.25 Hz; linear non-causal filter) to remove high-frequency artifacts, and high-passed filtered at 1 Hz (transition bandwidth 1 Hz; passband edge 1 Hz; cutoff frequency -6 dB 0.5 Hz; linear non-causal filter) to remove low-frequency drifts. 10–20 channel template locations from BESA spherical coordinates were used in *EEGLAB*. Artifactual channels (with ∼50% of data being noisy or artifactual) were manually tagged and removed with a custom-made single-page figure displaying each channel’s overall raw data, standard deviation, and power spectra. Files with at least one bad channel were removed for analyses.

An existing automatic method to clean EEG artifacts over this large sample was cross-validated: 150 files were randomly selected from the database to be cleaned manually and automatically with EEGLAB’s *clean_rawdata* plugin v2.2 (Euclidean method). Performance was calculated on each channel by comparing each sample as either true positive (TP, bad sample correctly rejected), true negative (TN, good sample correctly kept), false positive (FP, good sample incorrectly rejected), or false negative (FN, bad sample incorrectly kept). “Positive” and “negative” refer to presence or absence. Then, the true positive rate (TPR, i.e., sensitivity) and the true negative rate (TNR, i.e., specificity or selectivity) were calculated for each channel with: *TPR* = *TP/(TP* + *FN)* and *TNR* = *TN/(TN* + *FP).* The average sensitivity and specificity were then calculated over all channels to obtain the overall performance of the automatic method compared to manual rejection. After testing different parameters, the best performance obtained showed 81% sensitivity and 83% specificity [settings: *“burst_criteria”* = *6, “window_criteria”* = *0.3, “window_tolerance”* = *“(-Inf 7)*”]. 50 additional datasets were randomly selected for cross-validation, showing 84% sensitivity and 89% specificity. Since further increasing the sensitivity scores (i.e., removing more subtle artifacts) corresponded to a decrease in specificity (i.e., removing more non-artifactual data), these thresholds were considered most suited for this analysis. On average, this method removed an additional 11.4 s of data (± 23.0). Thus, bad channels were manually tagged and data were cleaned using this automated method and parameters. Files with less than 60 s of remaining artifact-free data were removed for analysis.

Note that this was done on duplicated data that were averaged-referenced to a fifth zero-filled channel as it increased performance by homogenizing raw signal amplitude across channels. But because this average re-referencing method was not validated for this specific montage and is not recommended with less than 30 channels (Smith et al., 2017), artifactual sections were removed from the original raw files and then re-processed as above. The issue of the electrode reference and its impact on asymmetry scores has been detailed and is of high importance (Allen et al., 2004; Smith et al., 2017). The recommended referencing methods (i.e., average-referencing, current-source density transformation) or the “residualization procedure” are not feasible with the low density and sparse montage of the Muse headset. The frontal channels are located close to the Fpz reference, potentially providing invalid asymmetry scores for the frontal channels by not reflecting the same underlying cortical activity as in the literature. Since frontal asymmetry estimated on linked-mastoid data is associated with the severity of current depression (Stewart et al., 2010), frontal channels were re-referenced to TP9/TP10. Temporoparietal channels were kept with the default Fpz reference.

#### Power Spectral Density and Asymmetry Estimates

Power Spectral Density (PSD) was calculated using MATLAB’s *pwelch* function on 1-s *hamming* tapered windows (42.5 dB sidelobe attenuation) with 50% overlap [per guidelines (Allen et al., 2004; Smith et al., 2017)], since the *pwelch* method smooths over non-systematic noise and is more robust compared to the more popular *fft* method that is more sensitive to noise and non-stationarities. Power spectra were then converted to 10*log10 deciBels (dB) as untransformed power values tend to be positively skewed due to individual differences in skull thickness that influence the signal amplitude (Allen et al., 2004).

The CoG was estimated for each channel using the automated, open-source method developed by Corcoran et al. (2017) which uses curve-fitting algorithms and a smoothing Savitzky-Golay Filter (SGF). This technique is thought to better account for interindividual variance and to be more reliable under low SNR conditions.

Asymmetry scores were obtained on the alpha PSD averaged over the predefined band (8–13 Hz), averaged over the predefined lower (8–10.5 Hz) and upper (11–13 Hz) sub-bands, and the individualized CoG.

They were calculated following standard procedures by subtracting the alpha power of interest of the left frontal channel from the right frontal channel (alpha_power_dB_AF8 – alpha_power_dB_AF7). Positive scores, therefore, indicate greater alpha power in the right relative to the left electrode. Asymmetry scores were also obtained from the temporoparietal (TP) channels. Finally, asymmetry scores were also computed on the delta (1–3 Hz), theta (4–7 Hz), and beta (14–30) frequency bands. Gamma was not included due to the Muse’s vulnerability to line noise in the high frequencies.

### Statistical Analyses

Robust linear regression models were generated in MATLAB 2021a using MATLAB’s *fitlm* package. Because of small portions of artifacts remaining in some EEG data after automatic preprocessing, robust least-squares regressions (Tukey’s bisquare function; default tuning constant = 4.685) were used for statistical analysis to down-weight the residuals’ influence on the model, using iterative reweighted least-squares (IRLS; Huber and Ronchetti, 2009). All models were tested for lack of fit first using a degenerate model consisting of only a constant term. Reported *F-statistics* with a *p-value*, therefore, indicate a valid fit for the model but do not inform on the relationship between the dependent and independent variables. The Beta (β) coefficient estimates and their standard error (SE) are reported in the first column and indicate a significant linear relationship between the predictor and the outcome variables when *p-values* are present. Summary statistics of the models include the number of observations, the error degrees of freedom, the root mean squared error (RMSE), R^2^ (for models with one predictor), adjusted R^2^ (for models with multiple predictors). Note that the descriptions below each table reporting the statistical results indicate whether the models were simple or multiple linear regressions (i.e., one or more predictor variables). In sum, all models were simple linear models and one was a multiple linear model (the two variables being lower and upper alpha asymmetry). Finally, following recommendations (Allen et al., 2004), asymmetry scores were also calculated on eleven 4-s blocks (as opposed to the average alpha power over all blocks for the asymmetry measures) to validate the internal reliability consistency of alpha asymmetry scores obtained on these short file lengths, using Cronbach’s alpha method, where a value below 0.2 indicates poor internal reliability consistency and greater than 0.8 a high internal reliability consistency (Cronbach, 1951).

## Results

230 participants remained for analyses after preprocessing. 83 files contained at least one bad channel and 36 had less than 60 s of artifact-free data and were excluded from the analyses (the data loss due to signal quality is discussed in the Discussion). They were aged from 22 to 80 years old (mean age was 55 ± 13.4) and were 64.3% female, 28.7% males, and 7% “Other” or missing. Cronbach’s alpha scores indicated a high internal reliability consistency of the asymmetry scores estimated on both frontal (Cronbach α = 0.95) and temporoparietal (Cronbach α = 0.82) channels.

### Well-Being and Alpha Asymmetry (Predefined Frequency Bands)

**FIGURE 1:**
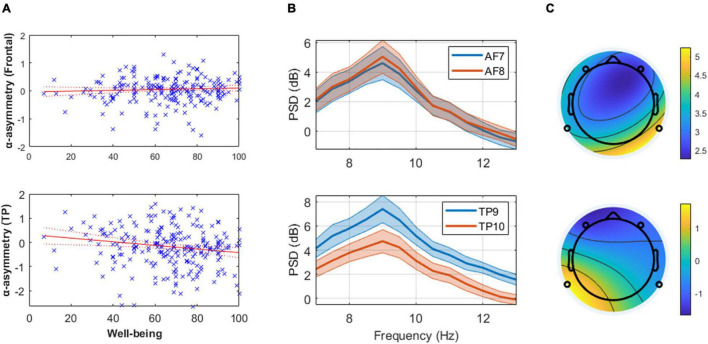
**(Panel A)** These linear regression models of well-being and mean alpha asymmetry (predefined 8–13 Hz band) show the absence of relationship at frontal channels (**top**) and the presence of one at temporoparietal (TP, **bottom**) channels. Higher well-being levels are associated with greater cortical activity in the right TP area relative to the left (assuming alpha inhibits regional cortical activity). **(Panel B)** Mean and standard error of the alpha power spectral density (PSD) from the 20 participants with highest reported well-being level at frontal **(top)** and TP **(bottom)** channels, illustrating the results reported in **(Panel A)**. **(Panel C)** Scalp topography of mean alpha PSD on a typical subject with low self-reported well-being (AIOS = 17; **top**) and high self-reported well-being (AIOS = 100; **bottom**), as an illustration of the effect reported in **(Panel A)**.

No association between subjective well-being levels and frontal alpha (predefined 8–13 Hz band) asymmetry was found (Figure 1 and Table 1). However, well-being was negatively correlated with TP alpha asymmetry scores (predefined 8–13 Hz band), reflecting greater cortical activity in the right TP area relative to the left is associated (assuming the inhibitory role of alpha oscillations on regional cortical activity; see Introduction). Detailed statistics are reported in Table 1 and an illustration of the results in the frequency and the scalp topography domain can be found in Figure 1, using the 20 participants with the highest well-being levels. The relationship between well-being and TP total alpha asymmetry scores appear to be driven more specifically by neural activity in the lower frequencies of the alpha band (8–10.5 Hz) because well-being was significantly correlated with lower alpha asymmetry but not with upper alpha asymmetry (see Table 2).

**TABLE 1:** Subjective well-being and alpha asymmetry (strict bounds at 8–13 Hz).

Predictor variable	β (SE)	N (DF)	Model RMSE	Model R^2^	Model F-statistic
Frontal α asymmetry	0.001 (0.002)	230 (228)	0.468	0.158	42.8***
TP α asymmetry	−0.007* (0.003)		0.808	0.036	8.51**

Column 2: p-values next to the Beta (β) coefficients and their standard error (SE) indicate a significant association between the predictor and the response variable at 95% confidence level (*), 99% confidence level (**) and 99.9% confidence level (***). Column 3: number of observations (N) and degrees of freedom (DF). Column 4–6: root mean square error (RMSE), R-squared, and F-statistic of the linear model. p-values next to F-statistic indicate a significant fit (see above for confidence levels). Each simple linear model follows the equation: Response variable ∼ 1 + predictor.

**TABLE 2:** Subjective well-being and temporoparietal (TP) lower/upper alpha asymmetry.

Predictor variable	Estimate (SE)	N (DF)	Model RMSE	Model R^2^	Model F-statistic
Lower α-asymmetry (8–10.5 Hz)	−0.008* (0.003)	230 (228)	0.981	0.035	8.28**
Upper α-asymmetry (11–13 Hz)	−0.005 (0.003)		0.863	0.011	2.61

Column 2: p-values next to the Beta (β) coefficients and their standard error (SE) indicate a significant association between the predictor and the response variable at 95% confidence level (*), 99% confidence level (**) and 99.9% confidence level (***). Column 3: number of observations (N) and degrees of freedom (DF). Column 4–6: root mean square error (RMSE), R-squared, and F-statistic of the linear model. p-values next to F-statistic indicate a significant fit (see above for confidence levels). The multiple linear model follows the equation: Response variable ∼ 1 + predictor1 + predictor2.

### Well-Being, Alpha Asymmetry (Predefined 8–13 Hz Band), and Covariates

**FIGURE 2:**
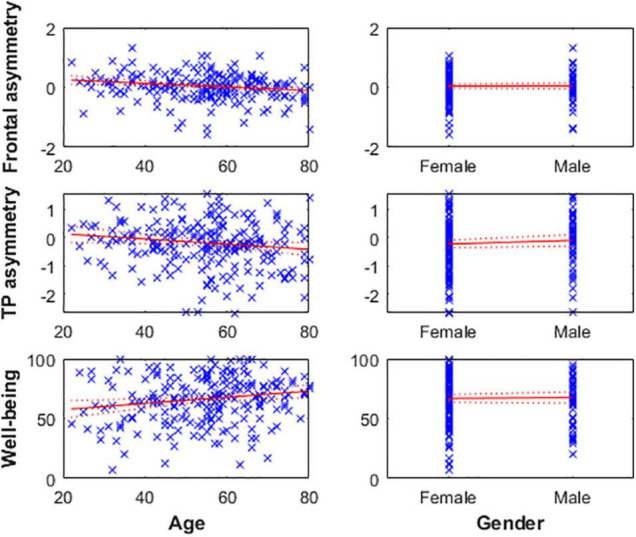
**Left:** Age is negatively associated with frontal **(top)** and TP **(middle)** alpha asymmetry scores, reflecting greater cortical activity in the right hemisphere relative to the left in older individuals. Age is positively associated with well-being levels **(bottom)**. **Right:** Gender was not associated with any of the three variables.

Age was negatively correlated with alpha asymmetry calculated on the predefined 8–13 Hz band (meaning the older the individual, the greater cortical activity is in the right frontal and TP areas relative to the left ones) and positively correlated with subjective well-being levels (i.e., older age reflecting greater well-being score). However, gender was not associated with well-being or alpha asymmetry (Figure 2 and Table 3).

**TABLE 3:** Subjective well-being and alpha asymmetry, and covariates.

Predictor variable	Estimate (SE)	N (DF)	Model RMSE	Model R^2^	Model F-statistic
**α-asymmetry (Frontal)**					
Age	−0.006* (0.002)	218 (216)	0.469	0.188	50***
Gender_Male	0.009 (0.071)	214 (212)	0.477	0.162	41***
**α-asymmetry (TP)**					
Age	−0.009* (0.004)	218 (216)	0.819	0.026	5.76*
Gender_Male	0.129 (0.123)	214 (212)	0.833	0.01	2.09
**Well-being**					
Age	0.258* (0.100)	218 (216)	19.7	0.031	7**
Gender_Male	0.68 (2.914)	214 (212)	19.7	0.003	0.56

Column 2: p-values next to the Beta (β) coefficients and their standard error (SE) indicate a significant association between the predictor and the response variable at 95% confidence level (*), 99% confidence level (**) and 99.9% confidence level (***). Column 3: number of observations (N) and degrees of freedom (DF). Column 4–6: root mean square error (RMSE), R-squared, and F-statistic of the linear model. p-values next to F-statistic indicate a significant fit (see above for confidence levels). Each simple linear model follows the equation: Response variable ∼ 1 + predictor.

### Well-Being, Alpha Center of Gravity, and Center of Gravity-Asymmetry

No linear relationships were observed between well-being and the CoG (Supplementary Table 2), and between well-being and asymmetry scores calculated on the CoG (Supplementary Table 3), for both frontal and TP channels.

### Well-Being and EEG Asymmetry in the Other Frequency Bands

No associations were observed between well-being and EEG asymmetry in the delta (1–3 Hz), theta (3–7 Hz), or beta (14–30 Hz) frequency bands (Supplementary Figure 1 and Supplementary Table 1).

## Discussion

### Results Summary

Contrary to the existing literature on the emotional valence and the motivational models of frontal EEG asymmetry, we found an absence of association between multidimensional well-being levels and frontal alpha asymmetry (predefined 8–13 Hz band, 8–10.5 Hz, and CoG-asymmetry). However, well-being was negatively correlated with alpha asymmetry at the TP sites (predefined 8–13 Hz and 8–10.5 Hz bands, but not for CoG-asymmetry), reflecting greater cortical activity in the right TP area relative to the left (assuming the inhibitory role of alpha oscillations on regional cortical activity; see Introduction). Interestingly, the direction of the asymmetry is opposite to the one in the frontal areas in the literature of frontal alpha asymmetry. Hence, while approach motivation and the related emotional processes are associated with relatively greater left than right frontal cortical activation, multidimensional well-being seems to be associated with asymmetric activation in the opposite direction in the TP areas.

This effect appears to be driven more specifically by oscillatory activity in the lower frequencies of the alpha band (8–10.5 Hz), aligning with studies highlighting the inhibitory function of these lower frequencies (Oakes, 2004). Making the distinction between lower and upper frequencies of the alpha band seems therefore especially relevant for neurophysiological studies using source-localization or simultaneous EEG-fMRI techniques to identify the intricate mechanisms involved in EEG asymmetry.

Contrary to our expectations, the CoG did not show associations with well-being levels. While CoG is associated with cognitive processes in the literature on the individual alpha frequency (IAF), we hypothesized that it would also be associated with self-reported well-being levels. However, the CoG may reflect other brain processes associated with cognition that are different than those involved with multidimensional well-being. Future studies using advanced source localization methods and high-density EEG systems should elucidate the different sources and networks associated with the different sub-components of alpha oscillations, and their associations with cognitive systems (i.e., PAF, CoG, lower/upper alpha).

While some researchers suspected that gender was the main driver of frontal alpha asymmetry levels (Gale et al., 2001; Dennis and Solomon, 2010; Mikolajczak et al., 2010), it was not associated with well-being or alpha asymmetry measures (for both frontal and temporoparietal sites) in this sample. However, age was negatively correlated with alpha asymmetry scores of both regions (meaning that cortical activity is greater in the right areas relative to the left ones as age increases) and positively correlated with subjective well-being levels. This finding aligns with the well-being literature (e.g., Carstensen et al., 2011), and supports a strong mediator role of age on the relationship between well-being and TP alpha asymmetry. Hence, the absence of a relationship between well-being and CoG-asymmetry might further indicate that there is a strong relationship between well-being, age, and alpha asymmetry in the TP area. Age is likely not the mechanism of change itself but may represent many underlying factors associated with brain changes and well-being (Kazdin, 2007). Thus, future studies using larger samples and higher density EEG data are necessary to confirm the accuracy of the asymmetry estimates obtained with this automated method, as well as to confirm or disprove the relationship between age, well-being, and alpha asymmetry in the TP area. If confirmed, the IAF-estimation method can be used to homogenize EEG asymmetry estimation procedures across investigators, and the specific interactions between these three variables should be further elucidated to determine the underlying mechanisms.

No associations were observed between subjective well-being and PSD asymmetry in the delta (1–3 Hz), theta (3–7 Hz), or beta (14–30 Hz) frequency bands (Supplementary Figure 1 and Supplementary Table 1), supporting the specific role of alpha oscillations in the brain processes underlying well-being.

### Interpretations of the Results and Potential Mechanisms

Studies using source-localization methods found the alpha asymmetry to originate mainly from brain activity in the dorsal system of the frontoparietal network (FPN; 13). Functional magnetic resonance imagery (fMRI) showed that this system is organized bilaterally and comprises the intraparietal sulcus (IPS) and the frontal eye fields (FEF) of each hemisphere, and is thought to mediate top-down guided voluntary allocation of attention to locations or features (Vossel et al., 2013). Both IPS and FEF are active when attention is overtly or covertly oriented in space and are suspected to be the regions for the maintenance of spatial priority maps, saccade planning, and visual working memory. In contrast, the ventral system comprises the temporoparietal junction (TPJ) and the ventral frontal cortex (VFC) and is associated with detecting unattended or unexpected stimuli and triggering shifts of attention (Vossel et al., 2013). It has been proposed that the ventral system is lateralized to the right hemisphere of the brain and exhibits asymmetric activity during attentional reorientation, the processing of rare deviant stimuli, and the response to valid vs. invalid cued targets (Corbetta and Shulman, 2002; Corbetta et al., 2008; Doricchi et al., 2010). The functional role of the TPJ also includes filtering irrelevant distractors during focused states of attention, modulating neural activity between various networks, and it has been implicated in social cognition and theory of mind (Vossel et al., 2013).

Hence, since our experimental task consisted of focusing attention on the breath, detecting mind-wandering thoughts (i.e., mental distractions), and reallocating attention to the goal, the TP alpha asymmetry may reflect these attentional processes and the underlying activity of the TPJ. Whereas, frontal alpha asymmetry may better reflect the dorsal system, as most studies use traditionally a cross-fixation task or resting-state condition with no focus of attention on any object. In line with these systems, one might speculate that participants with lower subjective well-being were more likely to ruminate on negative thoughts or memories (associated with negative valence and a withdrawal motivation; Mason et al., 2013; Smallwood and Andrews-Hanna, 2013) and less able to redirect their attention to their breath. This would decrease their capacity to detect negative thoughts and redirect their attention to their breath, corresponding to relatively greater left than right cortical activity in the TP area (positive TP asymmetry score). On the other hand, participants with higher well-being would be more likely to engage in mind wandering with positive valence and more likely to redirect their attention to their breath, which would correspond to greater cortical activity in the right TP area (negative TP asymmetry score). Another possibility is that alpha asymmetry in the TP regions might simply occur in opposite direction compared to the alpha asymmetry in the frontal areas (Davidson et al., 1990). Future studies using high-density systems and advanced source-localization methods are necessary to confirm or disprove this hypothesis.

### Limits and Recommendations

There are several limitations of this study that should be considered when reviewing the results.

While the AIOS-24h was found to be associated with longer-term well-being levels (i.e., reported well-being levels reflective of the past month and personality trait; see Methods), further validation is required to fully validate it as a measure of trait well-being.

While the asymmetry scores showed a relatively high internal reliability consistency and the Muse was validated for ERP research (Krigolson et al., 2017), 83 files had at least one bad channel and 36 had less than 60 s of remaining artifact-free data after preprocessing. This is a significant loss of data. The largest loss of data came from the presence of bad channels (considered bad when at least 50% of the channel was artifactual), likely due to the headband’s flexibility that is prone to moving and disconnecting electrodes. Thus, future investigators could consider using the more recent Muse S that was developed for sleep studies. The Muse S is made of a flexible fabric that can stretch and keep stronger pressure on the electrodes, preventing them from disconnecting as much. Furthermore, we recorded the data when participants already started the task with their eyes closed to reduce data cleaning over the large sample. Automatic cleaning performance would have likely been increased by adding a period before the task that includes obvious artifacts (e.g., asking participants to produce eye blinks and jaw clenching) to help the automatic method algorithms create a more robust baseline and therefore reject artifacts more efficiently. Thus, higher-grade and -density wearable EEG systems and longer recordings (at least 4 min of continuous data to ensure having at least 2 min of artifact-free data on a larger portion of the sample) are recommended for future studies to keep the advantages of wearable technologies to acquire large datasets without compromising data quantity and quality.

The Muse has only four channels. There are obvious benefits to having more EEG channels in terms of scalp distribution and data quality, which allow the use of advanced methods such as independent component analysis (ICA) which can be used to remove subtle artifacts such as muscle activity, subtle eye movements, or channel noise (Makeig et al., 1996; Delorme and Makeig, 2004). Furthermore, while we controlled for the potential reference issue using this system, a wearable headset with at least 30 channels would allow multiple referencing methods (e.g., average or CSD) and ensure highly accurate asymmetry estimates. However, this study showed that it is feasible to use a low-cost, low-density wearable system to examine the relationships between well-being and alpha asymmetry in a relatively large and diverse population.

Alpha center of gravity (CoG) and therefore CoG-asymmetry is expected to better account for interindividual differences. The automated IAF-estimation toolbox used in this study was not able to detect the CoG for 8 subjects (see Supplementary Tables 2, 3). We wanted to ensure that the absence of association between well-being and TP asymmetry calculated on the CoG was not due to this small sample difference (8 subjects missing compared to models on predefined alpha bands). Thus, we removed these 8 subjects from the model assessing the association between well-being and TP-asymmetry (predefined 8–13 Hz band) to see if the effect disappeared as a consequence of these 8 subjects being removed. Results showed that the significant association was still present (see Supplementary Table 4). Hence, this absence of association between well-being and CoG-asymmetry is either due to:

1) poorer estimation of alpha activity by the automatic method compared to the predefined band since the method performs best with more neighboring EEG channels (and the Muse has only four sparse channels). Here, we fed the algorithm with 2 channels at a time to avoid alpha contamination from distal channels (to keep alpha activity from frontal and TP channels separate).
2) this method better accounting for interindividual differences, which would indicate that the main effect (TP asymmetry calculated on the predefined 8–13 Hz band) might be a consequence of the relationship between age, well-being, and related brain activity.

Lastly, cross-sectional designs are always a limitation to consider. More sessions would be beneficial for the field to confirm the results and assess changes in both well-being and EEG asymmetry to evaluate the stability of this relationship over time.

### Long Term Applications and Goals

Attentional and inhibitory impairments are thought to be crucially associated with an increased vulnerability to depressive episodes and cognitive vulnerability (De Raedt and Koster, 2010). Alpha asymmetry (both frontal and TP) seems to play an essential role in understanding the neural networks underlying executive functions, attention, emotion regulation, and well-being. A better understanding of these processes is crucial to improving general well-being levels via targeted interventions. For example, Xu et al. (2018) found that positive psychological interventions (PPIs) increased not only subjective well-being and relief in depression but also left frontal asymmetry scores (Xu et al., 2018). Kim et al. (2012) found that positive reappraisals (i.e., techniques to recognize the negative pattern that one’s thoughts have taken using meta-awareness to cognitively reframe an event as more positive and therefore increase the sense of well-being) showed an increase in metabolic activity in the left dlPFC, caudate, and cingulate regions (Kim et al., 2012). Moynihan et al. (2013) found that mindfulness-based stress reduction produced significant changes in executive and immune functions, as well as in left frontal alpha asymmetry scores.

Neuroscientific tools such as neurofeedback (Linden, 2014; Brandmeyer and Delorme, 2020b) might increase these interventions’ efficacy by targeting brain networks on the same occasion. For instance, Angelakis et al. (2007) improved cognitive processing speed and executive function of elderly individuals using PAF as a neurofeedback index (Angelakis et al., 2007). Allen et al. (2001) found that increasing right frontal activity relative to the left using frontal asymmetry neurofeedback led to decreased positive affect (Allen et al., 2001).

Furthermore, neuromodulation techniques may be used to directly modulate specific networks such as the FPN. For example, some clinical studies have shown that exciting the left dlPFC with transcranial magnetic stimulation (TMS) or transcranial direct current stimulation (tDCS) improved depression symptoms (Kalu et al., 2012). Conversely, excitation of the right dlPFC led to reductions in craving (Boggio et al., 2008; Fregni et al., 2008) and risky decision-making (Fecteau et al., 2007), i.e., behaviors associated with difficulty in inhibiting extreme rewards with positive valence. Additionally, Sanguinetti et al. (2020) recently used novel transcranial focused ultrasound stimulation to target the right prefrontal cortex with higher resolution and depth than TMS or tDCS and successfully modulated mood and emotion regulation. By modulating both bottom-up and top-down systems, long-term solutions without side effects and at lower costs will emerge by helping patients self-control negative biases (Moser et al., 2002; Hanslmayr et al., 2011).

Understanding the role of third variables on these mechanisms will help adapt these therapies to meet each individual’s anatomy, physiology, and medical history, for more efficiency and safety. Once these intricacies are better understood, neuromodulation therapies might positively affect both the executive control and perceptive systems to decrease the propensity of depressive patients to focus on negative information and ruminative thought.

Finally, advancements in wearable technologies may allow care providers to monitor patients and apply neurofeedback or neuromodulation protocols at a low cost and remotely while patients are in the comfort of their homes (Cannard et al., 2020; Biondi et al., 2021).

## Conclusion

Overall, this study brings practical methodological information, challenges, and guidelines for conducting EEG research on large samples on well-being or related neuropsychological constructs, using wearable EEG technologies. Our findings bring novel knowledge that will help deepen our understanding of EEG asymmetries and their relations with well-being, the potential underlying neural networks and mechanisms, and the foreseeable long-term applications.

## Data Availability Statement

The datasets presented in this study can be found in online repositories. The names of the repository/repositories and accession number(s) can be found below: https://osf.io/nq7ga/.

## Ethics Statement

The studies involving human participants were reviewed and approved by Institute of Noetic Sciences’ Institutional Review Board. The patients/participants provided their written informed consent to participate in this study.

## Author Contributions

CC, HW, and AD made substantial contributions to the conception and design of the work, made substantial contributions to revising it critically for important intellectual content, and agreed to be accountable for all aspects of the work in ensuring that questions related to the accuracy or integrity of any part of the work are appropriately investigated and resolved. CC made a substantial contribution to the acquisition, analysis, interpretation of data, and writing the work. HW and AD provided approval for the publication of the content. All authors contributed to the article and approved the submitted version.

## Conflict of Interest

The authors declare that the research was conducted in the absence of any commercial or financial relationships that could be construed as a potential conflict of interest.

## Publisher’s Note

All claims expressed in this article are solely those of the authors and do not necessarily represent those of their affiliated organizations, or those of the publisher, the editors and the reviewers. Any product that may be evaluated in this article, or claim that may be made by its manufacturer, is not guaranteed or endorsed by the publisher.

## Footnotes

1
https://www.surveymonkey.com/
