# Supplementary material for: Electroencephalography Correlates of Well-Being Using a Low-Cost Wearable System
Source: Front Hum Neurosci. 2021 Dec 24;15:745135. doi: 10.3389/fnhum.2021.745135 (PMC8740323; doi:10.3389/fnhum.2021.745135)
Supplement: Supplementary file 1 [file Table_1.docx]

# SUPPLEMENTARY MATERIAL

**Table S1. Subjective well-being and asymmetry in other frequency bands.**

| **Independent variable** | **Estimate**  **(SE)** | **Number of observations (degrees of freedom)** | **Model**  **RMSE** | **Model**  **R^2^** | **Model F-statistic** |
| --- | --- | --- | --- | --- | --- |
| **Frontal asymmetry** | | | | | |
| **Delta (1-3 Hz)** | 0.001 (0.003) | 230 (228) | 0.99 | 0.0313 | 7.37** |
| **Theta (3-7 Hz)** | 0.001 (0.002) |  | 0.695 | 0.064 | 15.6*** |
| **Beta (14-30 Hz)** | 0.003 (0.003) |  | 0.851 | 0.135 | 32.4*** |
| **TP asymmetry** | | | | | |
| **Delta (1-3 Hz)** | 0.001 (0.003) | 230 (228) | 1.03 | 0.001 | 0.029 |
| **Theta (3-7 Hz)** | -0.003 (0.003) |  | 0.775 | 0.006 | 1.36 |
| **Beta (14-30 Hz)** | 0.001 (0.003) |  | 0.909 | 0.002 | 0.41 |

*p-values are reported with * (p-value < 0.05; significance at the 95% level), ** (p < 0.01; significance at the 99% level, and *** (p < 0.001; significance at the 99.9% level). p-values on the F-statistic indicate whether the model fit is valid or not, and p-values on the coefficient estimate that the linear relationship between the predictor and the response variables is significant. The standard deviation of the coefficients' distribution is reported in parentheses. Each simple linear model follows the equation: Response variable ~ 1 + predictor.*


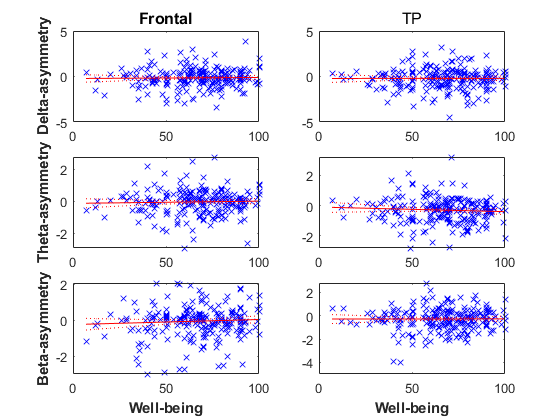


***Figure S1.*** *These linear regression models showed an absence of association between subjective well-being levels and* *PSD asymmetry in the Delta (1-3 Hz), Theta (3-7 Hz), and Beta (14-30 Hz) frequency bands.* ***Note****: Detailed statistics are reported in* ***Table S1****.*

**Table S2. Subjective well-being and alpha center of gravity (CoG).**

| **Independent variable** | **Estimate**  **(SE)** | **Number of observations (degrees of freedom)** | **Model**  **RMSE** | **Model**  **R^2^** | **Model F-statistic** |
| --- | --- | --- | --- | --- | --- |
| **Frontal α centre of gravity (CoG)** | 0.001 (0.004) | 222 (220) | 1.04 | 0.003 | 0.735 |
| **TP α centre of gravity (CoG)** | 0.002 (0.003) |  | 1.06 | 0.004 | 0.985 |

*p-values are reported with * (p-value < 0.05; significance at the 95% level), ** (p < 0.01; significance at the 99% level, and *** (p < 0.001; significance at the 99.9% level). p-values on the F-statistic indicate whether the model fit is valid or not, and p-values on the coefficient estimate that the linear relationship between the predictor and the response variables is significant. The standard deviation of the coefficients' distribution is reported in parentheses. Each simple linear model follows the equation: Response variable ~ 1 + predictor.*

**Table S3. Subjective well-being and CoG-asymmetry.**

| **Independent variable** | **Estimate**  **(SE)** | **Number of observations (degrees of freedom)** | **Model**  **RMSE** | **Model**  **R^2^** | **Model F-statistic** |
| --- | --- | --- | --- | --- | --- |
| **Frontal CoG-asymmetry** | -0.001 (0.002) | 222 (220) | 0.622 | 0.06 | 14.2*** |
| **TP CoG-asymmetry** | -0.007 (0.005) |  | 1.33 | 0.01 | 2.46 |

*p-values are reported with * (p-value < 0.05; significance at the 95% level), ** (p < 0.01; significance at the 99% level, and *** (p < 0.001; significance at the 99.9% level). p-values on the F-statistic indicate whether the model fit is valid or not, and p-values on the coefficient estimate that the linear relationship between the predictor and the response variables is significant. The standard deviation of the coefficients' distribution is reported in parentheses. Each simple linear model follows the equation: Response variable ~ 1 + predictor.*

**Table S4. Subjective well-being and TP-asymmetry, on the same sample as CoG-derived asymmetry (to check whether the absence of effect is due to the different sample or to poor estimations).**

| **Independent variable** | **Estimate**  **(SE)** | **Number of observations (degrees of freedom)** | **Model**  **RMSE** | **Model**  **R^2^** | **Model F-statistic** |
| --- | --- | --- | --- | --- | --- |
| **TP-asymmetry** | -0.007* (0.003) | 222 (220) | 0.808 | 0.028 | 6.33* |

*p-values are reported with * (p-value < 0.05; significance at the 95% level), ** (p < 0.01; significance at the 99% level, and *** (p < 0.001; significance at the 99.9% level). p-values on the F-statistic indicate whether the model fit is valid or not, and p-values on the coefficient estimate that the linear relationship between the predictor and the response variables is significant. The standard deviation of the coefficients' distribution is reported in parentheses. This simple linear model follows the equation: Response variable ~ 1 + predictor.*
